# Artificial Intelligence Used for Diagnosis in Facial Deformities: A Systematic Review

**DOI:** 10.3390/jpm14060647

**Published:** 2024-06-17

**Authors:** Victor Ravelo, Julio Acero, Jorge Fuentes-Zambrano, Henry García Guevara, Sergio Olate

**Affiliations:** 1 Grupo de Investigación de Pregrado en Odontología (GIPO), Universidad Autónoma de Chile, Temuco 4780000, Chile; victor.ravelo.s@gmail.com; 2 PhD Program in Morphological Science, Universidad de La Frontera, Temuco 4780000, Chile; 3 Department of Oral and Maxillofacial Surgery, Ramon y Cajal University Hospital, Ramon y Cajal Research Institute (IRYCIS), University of Alcala, 28034 Madrid, Spain; julio.acero@uah.es; 4 IT Managment Office, Universidad de La Frontera, Temuco 4780000, Chile; jfuentes3002@gmail.com; 5 Department of Oral Surgery, La Floresta Medical Institute, Caracas 1060, Venezuela; henryagg@gmail.com; 6 Division for Oral and Maxillofacial Surgery, Hospital Ortopedico Infantil, Caracas 1060, Venezuela; 7 Center for Research in Morphology and Surgery (CEMyQ), Universidad de La Frontera, Temuco 4780000, Chile; 8 Division of Oral, Facial and Maxillofacial Surgery, Universidad de La Frontera, Temuco 4780000, Chile

**Keywords:** artificial intelligence, machine learning, orthognathic surgery, diagnosis, face, morphology, facial deformity

## Abstract

AI is included in a lot of different systems. In facial surgery, there are some AI-based software programs oriented to diagnosis in facial surgery. This study aims to evaluate the capacity and training of models for diagnosis of dentofacial deformities in class II and class III patients using artificial intelligence and the potential use for indicating orthognathic surgery. The search strategy is from 1943 to April 2024 in PubMed, Embase, Scopus, Lilacs, and Web of Science. Studies that used imaging to assess anatomical structures, airway volume, and craniofacial positions using the AI algorithm in the human population were included. The methodological quality of the studies was assessed using the Effective Public Health Practice Project instrument. The systematic search identified 697 articles. Eight studies were obtained for descriptive analysis after exclusion according to our inclusion and exclusion criteria. All studies were retrospective in design. A total of 5552 subjects with an age range between 14.7 and 56 years were obtained; 2474 (44.56%) subjects were male, and 3078 (55.43%) were female. Six studies were analyzed using 2D imaging and obtained highly accurate results in diagnosing skeletal features and determining the need for orthognathic surgery, and two studies used 3D imaging for measurement and diagnosis. Limitations of the studies such as age, diagnosis in facial deformity, and the included variables were observed. Concerning the overall analysis bias, six studies were at moderate risk due to weak study designs, while two were at high risk of bias. We can conclude that, with the few articles included, using AI-based software allows for some craniometric recognition and measurements to determine the diagnosis of facial deformities using mainly 2D analysis. However, it is necessary to perform studies based on three-dimensional images, increase the sample size, and train models in different populations to ensure accuracy of AI applications in this field. After that, the models can be trained for dentofacial diagnosis

## 1. Introduction

The application of algorithms to learn and predict data using an artificial neural network has made it possible to address many layers in healthcare [1,2]. The scope of artificial intelligence (AI) enables clinical tasks to be performed with accuracy and less errors due to high precision, sensitivity, specificity, and accuracy in detection, disease classification, clinical decision-making [3], automated anatomical analysis, and the assessment and prediction of craniofacial growth and development [4].

Several AI-based software programs are used in maxillofacial surgery to process images (intraoral scans, 3D photographs, and tomographic images) for treatment planning and outcomes prediction [5]; however, clinical experience is needed to train machine learning based on craniomaxillofacial features and to corroborate the craniometric landmark or measurements, as well as the number and direction of hard and soft tissue movements required for the surgical treatment [6,7]. 

The use of deep learning image reconstruction algorithms, based on convolutional neural networks, improve the detection of anomalies, reducing radiation exposure and possibly creating medical imaging applications with precise diagnoses [8].

Cone beam computed tomography (CBCT) is a tool that provides three-dimensional volumetric data on maxillofacial structures and the assessment of airway area and volume [9]. The inclusion of AI for 3D image analysis enables the prediction of risk factors involved in diseases such as obstructive sleep apnea (OSA) syndrome [10], as well as speeding up the diagnosis and improving the data interpretation process [11,12]. 

The combination of CBCT and AI can help to find variables as predictors for the diagnosis of dentofacial deformities, and this can be the base for the indication for orthognathic surgery based on AI. Under controlled variables, some data show an 85–95.5% accuracy in their performance for dentofacial diagnosis [13]. Integrating AI technology with clinical evaluation and professional expert judgment can improve workflow and facilitate diagnostic and treatment procedures [14]. 

The face is used as a reliable biometric, because it is a unique marker [15] and allows for the determination of age, sex, ethnicity, and emotions, as well as structural characteristics and facial deformities [16]. Despite these benefits, the use of AI for facial analysis has a low frequency among clinicians due to the cost of facial scanners, which is why evaluations continue to be carried out based on the clinician’s experience [17].

This is an evolving topic, and this study aims to evaluate the evidence about the diagnosis of maxillofacial deformities using AI-based methods.

## 2. Materials and Methods

A systematic review was conducted per the Cochrane Handbook for Systematic Reviews of Interventions, and the report followed the updated Preferred Reporting Items for Systematic Reviews and Meta-Analyses (PRISMA) [18] to answer the following research question: can artificial intelligence be used to assess the diagnosis for orthognathic surgery in subjects with a CII or CIII skeletal facial deformity? (P: subjects with facial skeletal deformity CII and CIII; I: analyze skeletal position and airway with imaging; C: use artificial intelligence to analyze variables; O: determine the diagnosis of dentofacial deformities). We registered our protocol on PROSPERO, and the registration ID is as follows: 555053.

The search strategy was from 1943, which was the first published article on neuronal networks [19], to April 2024 using Medline, PubMed, Embase, Scopus, Lilacs, and Web of Science. There were no limitations on language or type of design. Studies published from 1945 onwards were selected, because the first paper on mathematical modeling for creating a neural network was published at that time. The terms included, using AND/OR, were “Artificial intelligence”, “computer-aided”, “deep learning”, “machine learning”, “neural networks”, “skeletal class”, and “facial morphology”. 

Two independent researchers carried out data selection. After search terms were applied, duplicates were removed. The remaining articles were reviewed using the Mendeley 2.90.0 software (Reference Management, Elsevier, London, UK). All articles were selected using the title and abstract, applying the inclusion and exclusion criteria. In case of discrepancy, a consensus was reached by discussion or consultation with a third reviewer.

Studies in English, Spanish, French, and Portuguese languages, presenting imaging studies to assess anatomical structures, airway volume, and craniofacial positions using an AI algorithm on a human population, and studies including measurements of datasets used to train, test, and validate AI models, as well as quantified measures of AI performance, were included. Secondary studies, case reports, reviews, and animal studies were excluded. 

Two reviewers extracted data and assessed the methodological quality of the studies using a pre-defined and standardized data form. A pilot test was used to ensure homogeneity of criteria among the reviewers. Reviewers were not blinded to the authors or journals.

(a) Study group data (number of patients, gender, age);
(b) Research data (prospective or retrospective nature of the study, dataset, AI architecture, validation of the AI method);
(c) Variables included and diagnoses (skeletal class, positions of bone structures, airway volume);
(d) Type of images (lateral teleradiography (2D) included, computed tomography (CT), cone beam computed tomography (CBCT), stereophotogrammetry (3D), and the software used in the analysis.

Two reviewers extracted data and assessed the methodological quality of the studies using a pre-defined and standardized data form. A pilot test was used to ensure homogeneity of criteria among reviewers. The reviewers were not biased toward authors or journals. 

The methodological quality of the studies was assessed using the Effective Public Health Practice Project (EPHPP) [20] instrument, which has the following six domains: selection bias, study design, confounders, blinding, data collection methods, and withdrawal and dropouts. Each methodological component was classified as strong, moderate, or weak based on the information provided by each study. The overall rating for each study was classified as strong when no component was weak, moderate when only one component was weak, and weak when two or more components were weak. 

## 3. Results

### 3.1. Article Selection

**Figure 1:**
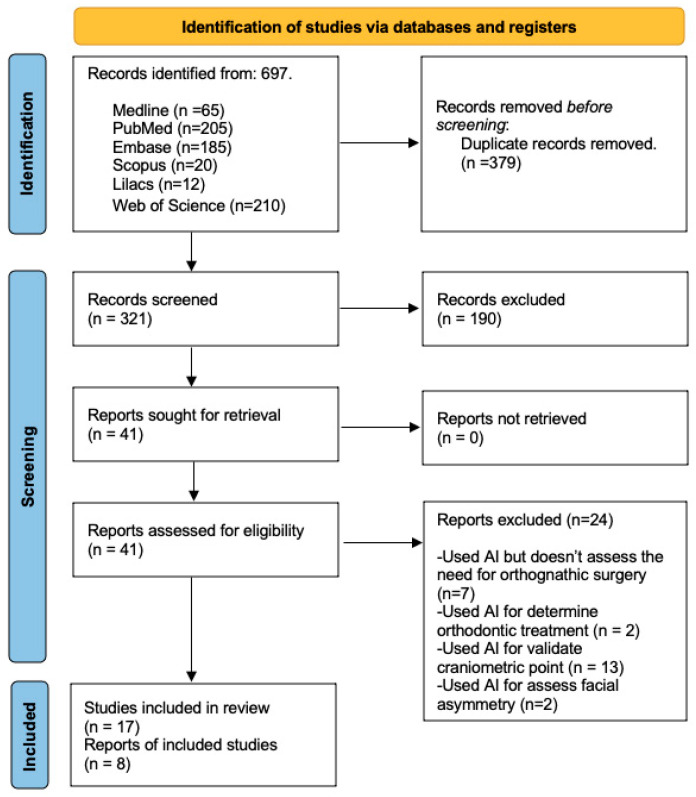
Flow chart of the systematic review.

The systematic search identified 697 articles. After excluding 379 duplicates, 321 articles were selected for title and abstract review, resulting in 17 articles for full-text review (Figure 1). Of the 17 articles, 4 studies were excluded, because their sample was less than 100 [21,22,23,24], and 5 studies were excluded, because their objectives did not include the facial diagnosis applied for orthognathic surgery using artificial intelligence, ultimately including 8 studies for descriptive and methodological analysis. 

### 3.2. Characteristics of the Included Studies

Of the five selected articles (Table 1), all studies were retrospective in design. A total of 5552 subjects were obtained, with an age range of 14.7 to years 56; 2474 (44.56%) subjects were male, and 3078 (55.43%) were female. Concerning ethnicity, three studies presented a sample in Korea, two on a population in China, and one on a population in the United States. 

Table 2 shows the descriptive results of the studies included in this research. At the diagnostic stage, two studies presented skeletal class II and class III subjects, two studies incorporated skeletal class III, and only the article by Shin et al. [28] incorporated subjects with facial asymmetry with class II and class III. Du et al. [31] mentions a lack of development of the maxilla or mandible, as well as if it presents deviation, while Xu et al. [32] only mentions whether there is the presence of mandibular retrognathism or prognathism. Only one study [27] does not mention the skeletal class of the sample, as it diagnoses them as needing (yes or no) orthognathic surgery. There was a higher frequency of subjects with skeletal class III, followed by skeletal class II, and only two studies identified skeletal class I subjects [29] and subjects with facial asymmetry [28]. All the studies used lateral cephalometry to identify the dentofacial morphology and the need for orthognathic surgery. Two studies [26,27] included in the diagnosis the use of clinical photographs, and one study [28] included frontal radiography. The software for the craniometric measurements and the analysis for orthognathic surgery differed in each study. Concerning the software for machine learning or deep learning, three studies [27,28,29] used Python software to process the results. Only Choi et al. [25] used the R software, while Khosravi-kamrani et al. [26], Taraji et al. [30], Du et al. [31], and Xu et al. [32] did not describe the software used to process the samples. The eight studies used different machine learning and deep learning models to process the data. 

Two studies [28,29] performed an analysis of class II and III skeletal patterns at the diagnostic stage to determine the need for orthognathic surgery. On other hand, Khosravi-kamrani et al. [26] and Taraji et al. [30] performed the same method for skeletal pattern validation, but only on skeletal class III subjects. Three studies [25,27,32] performed validations comparing subjects who were candidates for orthognathic surgery and those who did not need orthognathic surgery so that machine learning could find the algorithm more accurately. Two studies [27,28] used the convolutional neural network ResNet to process the data and evaluate which of all the processing methods was the most accurate. Li et al. [29] also used a convolutional neural network where DenseNet, in conjunction with stochastic gradient descent, made it possible to achieve greater accuracy. Only Choi et al. [25] described the use of a backward propagation network to train the neural network, and Khosravi-kamrani et al. [26] used a distance-weighted discrimination (DWD) method to perform the training. 

In terms of the imaging used for the analysis (Table 3), six studies used 2D imaging, all used different cephalometric software, and two studies [31,32] used 3D imaging for measurement and diagnosis, where only three studies [27,28,29] included the resolution and characteristics of the image used for the analyses. Regarding the parameters used for radiography, only one study [29] presented the milliampere, kilovoltage, and time ranges used during the radiation. Regarding measurements to determine the need for orthognathic surgery, only the study by Kim et al. [27] did not describe which analysis was used, as it was left to expert assessment. In contrast, the other studies used ANB angulation to determine the skeletal pattern. Choi et al. [25], Taraji et al. [30], Du et al. [31], and Xu et al. [32] used the ANB angle and the maxillary and mandibular discrepancy index as well as overjet and overbite measurements. Shin et al. [28] performed sagittal measurements such as ANB and Wits, while at the vertical level, the Jarabak and Björk index were used. On the other hand, only Khosravi-kamrani et al. [26] performed specific measurements of ANB angulation ≤ 0°, overjet ≤ 0 mm, and concave profile with an anterior inverted bite, because they only incorporated skeletal class III patients. 

All the studies obtained highly accurate results in facial diagnosis oriented to orthognathic surgery (Table 4). Four studies [27,28,29,32] using the convolutional neural network showed more than 80% prediction and accuracy. Choi et al. [25], Khosravi-kamrani et al. [26], and Taraji et al. [30], who used other machine learning algorithms, had 96–100% diagnostic accuracy. All the studies used sagittal patterns in relation to cephalometric parameters, while Shin et al. [28] and Du et al. [31] assessed the sensitivity and specificity of the results. 

### 3.3. Risk of Bias

**Figure 2:**
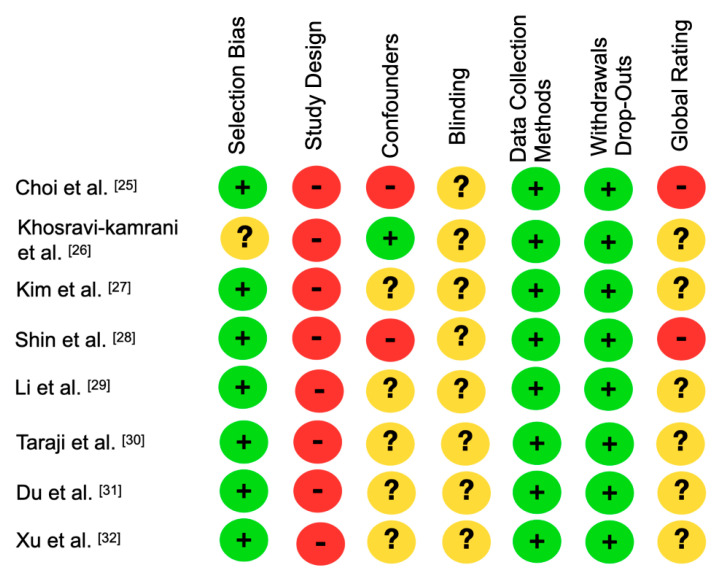
Summary of risk of bias of the included studies (green: strong; yellow: moderate; red: weak).

**Figure 3:**
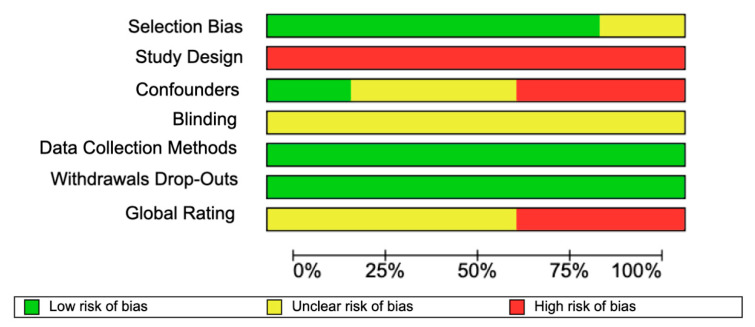
Summary plot risk of bias of the eight included studies.

The eight selected articles were assessed with the EPHPP tool (Figure 2). Regarding selection bias, seven studies had a strong assessment, while only one had a moderate assessment due to an unrepresentative sample. In the confounder item, two studies were high risk, because they used patients with facial deformity and asymmetry, whereas only one was low risk. In the blinding of the evaluators, all the studies had a moderate risk, because none mentioned whether patients were aware of the research. All eight studies had a low risk of bias in withdrawals or dropouts, as all studies reported completing the diagnostic and information processing stage in the neural network. The data collection and processing methods presented a strong assessment, as they all used artificial intelligence to process and train the data. Only in the study design did they have a weak evaluation. In the overall ranking (Figure 3), six studies were at moderate risk due to weak study designs, while two were at high risk of bias. The low risk of bias in five studies was due to high sample size, and data processing should be noted, as they were able to use a high sample size in both training and recognition of the need for orthognathic surgery.

## 4. Discussion

In 1943, McCulloch and Pitts published the first article on neural networks. Nowadays, convolutional neural networks (CNNs) are specialized artificial neural networks designed to process data from gridded structures, images, and videos to generate dimensional map learning to preserve and identify relevant information [33]. They can be used for image classification, target detection, segmentation, facial recognition, and medical image processing in different areas [34,35]. In contrast, PyTorch is a deep learning library that allows it to build, use, and connect various types of neural networks, enabling support for a wide range of tasks from natural language processing to computer vision [36]. 

The facial recognition algorithms started with methods that did not require geometric recognition of the face, but as their precision improved, local binary patterns and histograms of gradients oriented to facial features of the nose, eyes, and mouth were incorporated, until they reached the convolutional neural network and subsequent recognition of facial units and the identification of patterns that identify alterations or pathologies [16].

In our study, we identified two types of analyses, CNNs, and Pytorch. Although both are different composites, they can complement each other, because CNNs perform a neural network architecture to identify and filter (layer) visual tasks, while Pytorch performs deep learning model development to improve learning and performance. Studies using solely CNNs only performed craniometric-point identification and processing in conjunction with trained analyses and classifications. On the other hand, studies using CNNs and Pytorch with ResNet or DesNet models perform diagnosis and classification of parameters and can evolve through optimization techniques, such as stochastic gradient descent, which facilitates the creation and improvement of the neural network architecture. 

Regardless of the type of analysis, all the studies used ResNet or DesNet models, which differ in the depth of the network and the number and type of layers, as well as in the strategies used to improve their performance and efficiency during the training of the neural networks. Despite their differences, all networks were ultimately found to be over 80% accurate. Therefore, developing these models based on high-quality data makes diagnosing and evaluating pathologies in the maxillofacial region possible and provides important indicators that can guide surgery and postoperative management [37]. 

Both Lin et al. [38] and Lim et al. [39] found that AI can determine the diagnosis of facial deformities and also may be able to determine the need for orthognathic surgery using imaging variables such as cephalometry, growth patterns, and maxillomandibular rotations. The results reported in this review are highly accurate in their neural networks and in the use of cephalometric parameters; however, all these studies are also retrospective using two dimensions, and a three-dimensional analysis would deliver highly relevant information to the neural network [40]. 

Cheng et al. [21], in their study, proposed a neural network to define facial deformities and the potential use for orthognathic surgical needs; they showed the benefit for diagnosis, as well as for planning orthognathic surgery. In this study, they included an observation of maxillary movements and their effect on soft tissues. Ma et al. [22] used 3D imaging to perform a neural network framework to obtain skeletal references and predict postsurgical skeletal changes; the author obtained high accuracy and demonstrated the viability of predicting postoperative changes. Although soft tissue has not proven to be fully predictable due to its multiple presurgical and surgical technique variables [41], an accurate approach to soft tissue movements will be required in the next stages of AI development to help in orthognathic surgery planning and execution. Lo et al. [42] used a learning model with a convolutional neural network based on three-dimensional facial photographs to evaluate the presurgical and postsurgical assessments of facial structures in subjects treated with orthognathic surgery, showing that the neural network had significant improvements in terms of facial symmetry. 

Although all the studies had favorable results for the use of AI for diagnosis in dentofacial deformities, it should be noted that only 2D measurements were used to determine the diagnosis and the potential use for orthognathic surgical needs, and all the studies used different cephalometric analysis. Both the study by Shin et al. [28] and Li et al. [29] used sagittal measurements such as ANB and Wits to determine the maxillary positions, and only Shin et al. [28] included vertical measurements. The only study that incorporated sagittal, vertical, and transverse cephalometric skeletal measurements together with dental angulations was the study by Choi et al. [25]. Considering the analysis and the complexity of the face and the ratio between hard-to-soft tissue movement in orthognathic surgery, the only use of ANB as a strategy for diagnosis could be weak, and the association to orthognathic surgical needs would be lacking. A more complex analysis is necessary for facial diagnosis, as data can train the model, and after that, the AI can predict different strategies and variables for the final protocol.

Soft tissues remain an important factor in facial recognition, since, regardless of the method used, the thickness of facial soft tissue can mask skeletal alterations. Therefore, when reconstruction is performed using AI, it must include bone and soft tissues [8,43]. Alhazmi et al. [43] conducted a study of soft facial tissues between different sagittal skeletal patterns, observing that males with facial hypodivergence and class III show greater soft tissue thickness when compared to class I or class II skeletal conditions. 

Several authors [44,45,46] mention that there are differences in facial features when comparing ethnicity; some comparisons have shown similarities between 10.4% and 12.1% of facial features, while, when comparing subjects with European or American features, congruencies are observed between malar width and facial width at the level of the mandibular angle [47]. Gao et al. [48] made a comparison of facial aesthetics between Caucasian and Asian women, observing that Asian women have a small and less robust face, with a lower eyelid position and a rounder and smaller nose tip, as well as a retrognathic mandibular profile. In our research, it was noted that the population included was from Korea, China, and the United States, which limits the results of any analysis of AI associated to facial deformity diagnosis, because this bias can include age, ethnicity, gender, body mass index, and other variables with influence in the AI model, and this will be a key point in the next level of AI analysis in orthognathic surgery. 

The 2D image allows for sagittal measurements of maxillomandibular positions and the association to the skull base; 2D imaging is not used for quantitative values of airway or airway volume [49]. No study included in this analysis took measurements of airway volume to determine the need for orthognathic surgery. Using a systematic review, Neelapu et al. [50] mentioned that cephalometry provides important information on the anatomical bases that may influence airway analysis. Authors like Jayaratne and Zwahlen [51] showed that assessing airway area and 3D volume are necessary to quantitatively determine the association with skeletal patterns. On the other hand, Alhammadi et al. [52] evaluated the pharyngeal space of skeletal classes I, II, and III subjects using 3D imaging, observing that skeletal class II subjects have less airway volume than class I and class III subjects. For this reason, incorporating airway area and volume measurements along with skeletal class can provide important information for AI analysis and should be included in the facial diagnosis and in the recognition for facial surgery.

## 5. Conclusions

We can conclude that the use of AI-based software allows for craniometric surveys and measurements and could assist in dentofacial diagnosis; AI would predict diagnosis in different population settings and clinical conditions of facial deformity. It seems that the effort to produce good quality research in facial deformity diagnosis and orthognathic surgical needs shows a good standard using AI; however, regarding the overall analysis bias, three-dimensional analyses of the face are strongly needed, and new studies are a necessity.

## Figures and Tables

**Table 1:** Characteristics of the eight potential articles related to the study objective and patients included.

Author and Year	Objective	N	Sex (M/F)	Age (Years)
Chen et al. [25]	Develop a new artificial intelligence model for dentofacial diagnosis and orthognathic surgery indicating decision-making using neural network machine learning.	316	123–193	ND
Khosravi-kamrani et al. [26]	Use a new statistical prediction model to assess skeletal class III subjects and their need for orthognathic surgery.	148	68–80	14–25
Kim et al. [27]	Investigate the relationship between cephalometric imaging patterns and the need for orthognathic surgery using neural network predictive models.	960	468–492	24.6 (±4.9)
Shin et al. [28]	Develop a deep learning network to predict the facial morphology and the need for orthognathic surgery automatically.	840	461–379	23.2 (19–29)
Li et al. [29]	Compare the performance of different convolutional neural network algorithms to classify skeletal patterns and identify when they are class II and class III.	2431	1018–1413	25.5 (12–42)
Taraji et al. [30]	The aim is to identify critical morphological features in postcircumpubertal Cl III treatment and appraise the predictive ability of innovative machine learning (ML) algorithms for adult Cl III malocclusion treatment planning.	182	91–91	16 and 29
Du et al. [31]	The present study created an interactive decision support system that could output an accurate diagnosis of dentomaxillofacial deformities and recommend individual surgical plans based on surgeon preferences	574	203–371	23.4 (±7.2) and 26.3 (±8.5)
Xu et al. [32]	The objectives were development a machine learning model for diagnosing mandibular retrognathism and prognathism and compare the performance of the developed machine learning model	101	42–59	14 to 56

Obs: N: number; M: male; F: female ND: not described.

**Table 2:** Descriptive analysis of the articles included in the diagnosis of dentofacial deformity using artificial intelligence.

Author and Year	Ethnicity	Study Design	Diagnosis	Software for Craniometric Measurements	2D or 3D Imaging	Software for Machine Learning or Deep Learning	Machine Learning or Deep Learning Model	Method of Measurement
Choi et al. [25]	Korean	Retrospective	Skeletal class II and III	V-ceph program (version 5.3 Osstem Inc., Seoul, Republic of Korea)	Lateral cephalometry	R project for statistical computing	Two-layer neural network with one hidden layer and four hidden nodes in the hidden layer	Calculated by comparing the real diagnosis with the diagnosis obtained by the artificial intelligence model.
Khosravi-kamrani et al. [26]	United States	Retrospective	Skeletal class III	Dolphin imaging software	Lateral cephalometry and clinical photography	ND	Distance weighted discrimination (DWD) method	The statistical prediction method was used in mandibular prognathism, deficient maxilla, and a combination of the two.
Kim et al. [27]	Korea	Retrospective	Needed orthodontic treatments and needed orthognathic surgery	WebCeph program (assemble circle, Seul, Korea)	Lateral cephalometry and clinical photography	Python Keras and Tensorflow backend engine.	Convolutional neural network (CNN) models ResNet-18, ResNet-34, ResNet-50, and ResNet-101.	The orthognathic surgery diagnoses were validated, and the algorithm with the best prediction (99.86%) was trained.
Shin et al. [28]	Korea	Retrospective	Skeletal class II and III, facial asymmetry	Planmeca Promax (Planmeca OY, Helsinki, Finland)	Lateral cephalometry and frontal radiography	PyTorch (Python Software)	Backbone feature extraction used ResNet34, with hierarchically stacked convolution blocks.	Feature extraction is performed on each image and then merged.
Li et al. [29]	China	Retrospective	Skeletal class I, II, and III	Veraviewepocs 2D (J Morita Corp., Kyoto, Japan)	Lateral cephalometry	PyTorch (Python Software)	All convolutional neural network (CNN) layers were trained using stochastic gradient descent (SGD) fine-tuning techniques, with DenseNet 161 being the most accurate.	A skeletal pattern extraction was performed to identify skeletal Class I, II, and III subjects.
Taraji et al. [30]	The racial composition of the groups varied.	Retrospective	Skeletal III	Dolphin Imaging program (Windows, Version 11.95: Chatsworth, CA, USA)	Lateral cephalometry and clinical photography	ND	Support Vector Machine, Multi- layer Perceptron (MLP), k-Nearest Neighbor, Random Forest, Convolutional Neural Network and Extreme Gradient Boosting.	The XGBoost classifiers achieved 100% specificity rates when predicting camouflage treatment. XGBoost was most sensitive for the surgical cohort.
Du et al. [31]	China	Retrospective	Maxillary development, mandibular development, maxillary deviation, and mandibular deviation.	Mimics 16.0 software (Materialise Inc., Leuven, Belgium)	Spiral computerized tomography and clinical photography	ND	BR-XGBoost, neural networks, and support vector machines algorithm.	The diagnostic model classified maxillofacial deformities diagnosis and the output results contain six 3D parameters representing surgery planification of rotation and movement of maxilla, mandible, and chin
Xu et al. [32]	ND	Retrospective	Diagnosis of the mandibular anteroposterior position was made (a) normal, (b) retrognathism, (c) prognathism.	AnatomicAligner System for Surgical Planning (Houston Methodist Research Institute, Houston, TX, USA)	3D facial scanner and computed tomography	ND	A seven-layer multilayer perceptron	diagnostic tests used to diagnose mandibular anteroposterior position: SNB angle, facial angle, mandibular unit length for mandibular anteroposterior position ate

Obs: ND: not described; 2D: two-dimensional; 3D: three-dimensional.

**Table 3:** Image characteristics included in AI analysis.

Author and Year	Imaging Equipment	Parameters for Image Acquisition	Analysis to Determine the Facial Diagnosis	Image Format
Choi et al. [25]	ND	ND	ANB and dentition angulation; maxillary and mandibular discrepancy index; overjet and protrusion.	ND
Khosravi-kamrani et al. [26]	ND	ND	ANB ≤ 0°; Overjet ≤ 0 mm; concave profile with anterior inverted bite	ND
Kim et al. [27]	ND	ND	ND	The image was resized to 256 × 256 pixels.
Shin et al. [28]	Planmeca, Helsinki, Finland	ND	ANB and Wits angulation for determining sagittal skeletal relationship. The Jarabak and Björk index was used to determine the vertical ratio.	The image had a pixel resolution of 2045 × 1816.
Li et al. [29]	Veraviewepocs 2D (J Morita Corp, Kyoto, Japan)	time, 4.9 s; tube current, 5–10 mA; tube voltage, 90 kV	Cephalometric measurements to determine skeletal class: skeletal class I pattern (5° ≥ ANB ≥ 0° and 2 ≥ Wits ≥ −3), skeletal class II pattern (ANB > 5° and Wits > 2), and skeletal class III pattern (ANB < 0° and Wits < −3)	JPG 224 × 224 pixels using the OpenCV package
Taraji et al. [30]	ND	ND	ANB and dentition angulation; maxillary and mandibular discrepancy index; overjet and overbite.	ND
Du et al. [31]	ND	ND	Measurements to skeletal class, SNA, SNB, SNPog and maxillary and mandibular discrepancy angulations	ND
Xu et al. [32]	ND	ND	Mandibular anteroposterior position: SNB angle, facial angle and mandibular unit length.	ND

**Table 4:** Characteristics of the measurement method and results of the articles included for diagnosis in dentofacial deformity using artificial intelligence.

Author and Year	Method of Measurement	Main Results
Choi et al. [25]	2D lateral cephalometric craniometric points were measured on class II and III subjects. A neural network was used.	Machine learning obtained between 96 and 100% to confirm diagnosis. Validation to recognize class II and class III subjects who were candidates for orthognathic surgery.
Khosravi-kamrani et al. [26]	Craniometric point measurements of 2D lateral cephalometry and photographs in skeletal class III subjects, using the statistical prediction method in mandibular prognathism, deficient maxilla, and a combination of the two.	The model was most effective in predicting subjects with mandibular prognathism, followed by maxillary deficiency, and finally, a combination of the two, despite being more difficult to diagnose in some classifications.
Kim et al. [27]	Using 2D radiographic analysis, clinical examination, and clinical photography, subjects who were candidates for orthognathic surgery and subjects who did not need surgery were included.	The facial diagnosis of patients get prediction in 97.85% and the data could be used for orthognathic surgical needs.
Shin et al. [28]	Craniometric points from lateral cephalometric and frontal radiographs is performed	The results showed high sensitivity and specificity rates (0.9554, 0.844, and 0.993) for craniometric measurements to assess facial diagnosis and potentially orthognathic surgical needs.
Li et al. [29]	Using 2D radiographic analysis, and craniometric measurements were included to find the skeletal class.	Convolutional neural networks identified sagittal patterns in the lateral cephalometric parameters. Accuracy was highest in class III subjects (97%), followed by class II (93%), and lastly by class I (87%).
Taraji et al. [30]	Using 2D radiographic analysis and clinical photography, encompassed subjects’ skeletal class III who underwent orthognathic surgery or camouflage mechanotherapy.	Wits analysis, ANB angulation and mandibular plane angulation significantly affected determining whether camouflage or orthognathic surgery is necessary. There was a diagnostic accuracy of 91 to 93% to determine whether a CIII subject would undergo orthodontic camouflage or orthognathic surgery.
Du et al. [31]	Using extraoral and intraoral photographs, and measurements craniometric position for diagnostic maxillo-mandibular overdevelopment and/or deviation for planification surgery orthognathic.	The diagnostic model classified the dentomaxillofacial deformities and the combination of the two provided the final diagnosis. The algorithm showed the highest accuracy and sensitivity of 0.881 to 0.9282 for classification of different types of dentomaxillofacial deformities.
Xu et al. [32]	Presurgical computed tomography and 3D scan images were used to perform mandibular anteroposterior measurements and compare the diagnosis by algorithm, a software to determine the need for surgery and an experienced surgeon as a gold standard.	The algorithm can accurately diagnose jaw deformities using 3D landmarks, demonstrating performance beyond that of traditional cephalometric measurements with a diagnostic accuracy of 85.2%.

Obs: 2D: two-dimensional; 3D: three-dimensional.

## Data Availability

The data are available upon request from the corresponding author.

## References

[1] Ramesh A.N., Kambhampati C., Monson J.R., Drew P.J. (2004). Artificial intelligence in medicine. Ann. R. Coll. Surg. Engl..

[2] Schwendicke F., Samek W., Krois J. (2020). Artificial intelligence in dentistry: Chances and challenges. J. Dent. Res..

[3] Ahmed N., Abbasi M.S., Zuberi F., Qamar W., Halim M.S.B., Maqsood A., Alam M.K. (2021). Artificial intelligence techniques: Analysis, application, and outcome in dentistry a systematic review. Biomed. Res. Int..

[4] Ossowska A., Kusiak A., Świetlik D. (2022). Artificial intelligence in dentistry-narrative review. Int. J. Environ. Res. Public Health.

[5] Rokhshad R., Keyhan S.O., Yousefi P. (2023). Artificial intelligence applications and ethical challenges in oral and maxillo-facial cosmetic surgery: A narrative review. Maxillofac. Plast. Reconstr. Surg..

[6] Mohaideen K., Negi A., Verma D.K., Kumar N., Sennimalai K., Negi A. (2022). Applications of artificial intelligence and machine learning in orthognathic surgery: A scoping review. J. Stomatol. Oral Maxillofac. Surg..

[7] Douglas M.J., Callcut R., Celi L.A., Merchant N. (2023). Interpretation and use of applied/operational machine learning and artificial intelligence in surgery. Surg. Clin. N. Am..

[8] Chandran M.O., Pendem S.P.S.P., Chacko C.P., Kadavigere R. (2024). Influence of deep learning image reconstruction algorithm for reducing radiation dose and image noise compared to iterative reconstruction and filtered back projection for head and chest computed tomography examinations: A systematic review. F1000Research.

[9] Abesi F., Maleki M., Zamani M. (2023). Diagnostic performance of artificial intelligence using cone-beam computed tomography imaging of the oral and maxillofacial region: A scoping review and meta-analysis. Imaging Sci. Dent..

[10] Shujaat S., Jazil O., Willems H., Van Gerven A., Shaheen E., Politis C., Jacobs R. (2021). Automatic segmentation of the pharyngeal airway space with convolutional neural network. J. Dent..

[11] Badr F.F., Jadu F.M. (2022). Performance of artificial intelligence using oral and maxillofacial CBCT images: A systematic review and meta-analysis. Niger. J. Clin. Pract..

[12] Tsolakis I.A., Kolokitha O.E., Papadopoulou E., Tsolakis A.I., Kilipiris E.G., Palomo J.M. (2022). Artificial intelligence as an aid in CBCT airway analysis: A systematic review. Life.

[13] Huqh M.Z.U., Abdullah J.Y., Wong L.S., Jamayet N.B., Alam M.K., Rashid Q.F., Husein A., Ahmad W.M.A.W., Eusufzai S.Z., Prasadh S. (2022). Clinical applications of artificial intelligence and machine learning in children with cleft lip and palate—A systematic review. Int. J. Environ. Res. Public Health.

[14] Wong K.F., Lam X.Y., Jiang Y., Yeung A.W.K., Lin Y. (2023). Artificial intelligence in orthodontics and orthognathic surgery: A bibliometric analysis of the 100 most-cited articles. Head Face Med..

[15] Kaur P., Krishan K., Sharma S.K., Kanchan T. (2020). Facial-recognition algorithms: A literature review. Med. Sci. Law.

[16] Qiang J., Wu D., Du H., Zhu H., Chen S., Pan H. (2022). Review on facial-recognition-based applications in disease diagnosis. Bioengineering.

[17] Patcas R., Bornstein M.M., Schätzle M.A., Timofte R. (2022). Artificial intelligence in medico-dental diagnostics of the face: A narrative review of opportunities and challenges. Clin. Oral Investig..

[18] Page M.J., McKenzie J.E., Bossuyt P.M., Boutron I., Hoffmann T.C., Mulrow C.D., Shamseer L., Tetzlaff J.M., Akl E.A., Brennan S.E. (2021). The PRISMA 2020 statement: An updated guideline for reporting systematic reviews. BMJ.

[19] McCulloch W.S., Pitts W. (1943). A logical calculus of the ideas immanent in nervous activity. Bull. Math. Biophys..

[20] Thomas B.H., Ciliska D., Dobbins M., Micucci S. (2004). A process for systematically reviewing the literature: Providing the research evidence for public health nursing interventions. Worldviews Evid. Based Nurs..

[21] Cheng M., Zhang X., Wang J., Yang Y., Li M., Zhao H., Huang J., Zhang C., Qian D., Yu H. (2023). Prediction of orthognathic surgery plan from 3D cephalometric analysis via deep learning. BMC Oral Health.

[22] Ma Q., Kobayashi E., Fan B., Hara K., Nakagawa K., Masamune K., Sakuma I., Suenaga H. (2022). Machine-learning-based approach for predicting postoperative skeletal changes for orthognathic surgical planning. Int. J. Med. Robot..

[23] Deng H.H., Liu Q., Chen A., Kuang T., Yuan P., Gateno J., Kim D., Barber J.C., Xiong K.G., Yu P. (2023). Clinical feasibility of deep learning-based automatic head CBCT image segmentation and landmark detection in computer-aided surgical simulation for orthognathic surgery. Int. J. Oral Maxillofac. Surg..

[24] Tao L., Li M., Zhang X., Cheng M., Yang Y., Fu Y., Zhang R., Qian D., Yu H. (2023). Automatic craniomaxillofacial landmarks detection in CT images of individuals with dentomaxillofacial deformities by a two-stage deep learning model. BMC Oral Health.

[25] Choi H.I., Jung S.K., Baek S.H., Lim W.H., Ahn S.J., Yang I.H., Kim T.W. (2019). Artificial intelligent model with neural network machine learning for the diagnosis of orthognathic surgery. J. Craniofac. Surg..

[26] Khosravi-Kamrani P., Qiao X., Zanardi G., Wiesen C.A., Slade G., Frazier-Bowers S.A. (2022). A machine learning approach to determine the prognosis of patients with Class III malocclusion. Am. J. Orthod. Dentofacial Orthop..

[27] Kim Y.H., Park J.B., Chang M.S., Ryu J.J., Lim W.H., Jung S.K. (2021). Influence of the depth of the convolutional neural networks on an artificial intelligence model for diagnosis of orthognathic surgery. J. Pers. Med..

[28] Shin W., Yeom H.G., Lee G.H., Yun J.P., Jeong S.H., Lee J.H., Kim H.K., Kim B.C. (2021). Deep learning based prediction of necessity for orthognathic surgery of skeletal malocclusion using cephalogram in Korean individuals. BMC Oral Health.

[29] Li H., Xu Y., Lei Y., Wang Q., Gao X. (2022). Automatic classification for sagittal craniofacial patterns based on different convolutional neural networks. Diagnostics.

[30] Taraji S., Atici S.F., Viana G., Kusnoto B., Allareddy V.S., Miloro M., Elnagar M.H. (2023). Novel machine learning algorithms for prediction of treatment decisions in adult patients with class III malocclusion. J. Oral Maxillofac. Surg..

[31] Du W., Bi W., Liu Y., Zhu Z., Tai Y., Luo E. (2024). Machine learning-based decision support system for orthognathic diagnosis and treatment planning. BMC Oral Health.

[32] Xu X., Deng H.H., Kuang T., Kim D., Yan P., Gateno J. (2024). Machine learning effectively diagnoses mandibular deformity using three-dimensional landmarks. J. Oral Maxillofac. Surg..

[33] Nagendran M., Chen Y., Lovejoy C.A., Gordon A.C., Komorowski M., Harvey H., Topol E.J., Ioannidis J.P.A., Collins G.S., Maruthappu M. (2020). Artificial intelligence versus clinicians: Systematic review of design, reporting standards, and claims of deep learning studies. BMJ.

[34] Zhou L.Q., Wang J.Y., Yu S.Y., Wu G.G., Wei Q., Deng Y.B., Wu X.L., Cui X.W., Dietrich C.F. (2019). Artificial intelligence in medical imaging of the liver. World J. Gastroenterol..

[35] Gore J.C. (2020). Artificial intelligence in medical imaging. Magn. Reson. Imaging.

[36] Pazke A., Gross S., Massa F., Lerer A., Bradbury J., Chanan G., Killen T., Zeming L., Gimelshein N., Antiga L. PyTorch: An Imperative Style, High-Performance Deep Learning Library. Proceedings of the 33rd Conference on Neural Information Processing Systems (NeurIPS).

[37] Rekawek P., Rajapakse C.S., Panchal N. (2021). Artificial Intelligence: The future of maxillofacial prognosis and diagnosis?. J. Oral Maxillofac. Surg..

[38] Lin G., Kim P.J., Baek S.H., Kim H.G., Kim S.W., Chung J.H. (2021). Early Prediction of the need for orthognathic surgery in patients with repaired unilateral cleft lip and palate using machine learning and longitudinal lateral cephalometric analysis data. J. Craniofac. Surg..

[39] Lim J., Tanikawa C., Kogo M., Yamashiro T. (2021). Determination of prognostic factors for orthognathic surgery in children with cleft lip and/or palate. Orthod. Craniofac. Res..

[40] Wu T.Y., Lin H.H., Lo L.J., Ho C.T. (2017). Postoperative outcomes of two- and three-dimensional planning in orthognathic surgery: A comparative study. J. Plast. Reconstr. Aesthet. Surg..

[41] Olate S., Zaror C., Mommaerts M.Y. (2017). A systematic review of soft-to-hard tissue ratios in orthognathic surgery. Part IV: 3D analysis—Is there evidence?. J. Craniomaxillofac. Surg..

[42] Lo L.J., Yang C.T., Ho C.T., Liao C.H., Lin H.H. (2021). Automatic Assessment of 3-dimensional facial soft tissue symmetry before and after orthognathic surgery using a machine learning model: A preliminary experience. Ann. Plast. Surg..

[43] Alhazmi N., Alrasheed F., Alshayea K., Almubarak T., Alzeer B., Alorf M.S., Alshanqiti A., Albalawi M. (2023). Facial soft tissue characteristics among sagittal and vertical skeletal patterns: A cone-beam computed tomography study. Cureus.

[44] Kau C.H., Richmond S., Zhurov A., Ovsenik M., Tawfik W., Borbely P., English J.D. (2010). Use of 3-dimensional surface acquisition to study facial morphology in 5 populations. Am. J. Orthod. Dentofacial Orthop..

[45] Wirthlin J., Kau C.H., English J.D., Pan F., Zhou H. (2013). Comparison of facial morphologies between adult Chinese and Houstonian Caucasian populations using three-dimensional imaging. Int. J. Oral Maxillofac. Surg..

[46] Wen Y.F., Wong H.M., Lin R., Yin G., McGrath C. (2015). Inter-ethnic/racial facial variations: A systematic review and bayesian meta-analysis of photogrammetric studies. PLoS ONE.

[47] Islam S., Taylor C.J., Hayter J.P. (2017). Analysis of facial morphology of UK and US general election candidates: Does the ‘power face’ exist?. J. Plast. Reconstr. Aesthet. Surg..

[48] Gao Y., Niddam J., Noel W., Hersant B., Meningaud J.P. (2018). Comparison of aesthetic facial criteria between Caucasian and East Asian female populations: An esthetic surgeon’s perspective. Asian J. Surg..

[49] Ravelo V., Olate G., Muñoz G., de Moraes M., Olate S. (2021). The airway volume related to the maxillo-mandibular position using 3d analysis. Biomed. Res. Int..

[50] Neelapu B.C., Kharbanda O.P., Sardana H.K., Balachandran R., Sardana V., Kapoor P., Gupta A., Vasamsetti S. (2017). Craniofacial and upper airway morphology in adult obstructive sleep apnea patients: A systematic review and meta-analysis of cephalometric studies. Sleep Med. Rev..

[51] Jayaratne Y.S.N., Zwahlen R.A. (2016). The oropharyngeal airway in young adults with skeletal class II and class III deformities: A 3-D morphometric analysis. PLoS ONE.

[52] Alhammadi M.S., Almashraqi A.A., Halboub E., Almahdi S., Jali T., Atafi A., Alomar F. (2021). Pharyngeal airway spaces in different skeletal malocclusions: A CBCT 3D assessment. Cranio.

